# Serous retinal detachment as an early manifestation of lupus choroidopathy

**DOI:** 10.5935/0004-2749.20210097

**Published:** 2025-08-21

**Authors:** Mariela Regina Dalmarco Ghem, Anne Caroline Hungaro, Kenzo Hokazono

**Affiliations:** 1 Department of Ophthalmology, Universidade Federal do Paraná, Curitiba, PR, Brazil

**Keywords:** Lupus erythematosus, systemic, Retinal detachment, Lúpus eritematoso sistêmico, Descolamento da retina

## Abstract

Serous retinal detachment can be caused by a wide range of conditions, including systemic lupus erythematosus. Although this association has been well-described in patients with an established diagnosis of systemic lupus erythematosus, in rare cases, this detachment is the initial manifestation. We have described here an unusually challenging case in which serous retinal detachment required a comprehensive investigation considering that it was an early sign of systemic lupus erythematosus.

## Abstract (pt)

Descolamento seroso de retina pode ser causado por uma variedade de patologias, incluindo o lúpus eritematoso sistêmico. Embora essa associação seja bem estabelecida em pacientes com diagnóstico prévio de lúpus eritematoso sistêmico, o descolamento seroso de retina raramente é a manifestação inicial. Descrevemos um caso incomum e desafiador, o qual demandou ampla investigação por ter sido o descolamento seroso de retina a manifestação inicial do lúpus eritematoso sistêmico.

## INTRODUCTION

Systemic lupus erythematosus (SLE) is an autoimmune disease of unknown etiology that involves multiple organs and frequently affects the eyes. It is diagnosed when at least 4 of 11 criteria are present or were present sometime in the past, as established by the American College of Rheumatology^(1)^. The criteria include malar rash, discoid rash, photosensitivity, oral ulcers, non-erosive arthritis, serositis, renal disorder, neurological disorder, hematological disorder, immunological disorder, and the presence of antinuclear antibodies. Ocular involvement occurs in approximately one-third of all patients and involves the abnormalities of eyelids, keratoconjunctivitis sicca, uveitis, retinal and choroidal disorder, scleritis, and optic neuropathy^(2)^. Most commonly in practice, these ophthalmic findings are assigned to SLE in patients with a confirmed diagnosis. However, since almost every ocular structure can be affected, high diagnostic suspicion is required for patients without other systemic manifestations. We have presented here a case in which the choroidopathy was the most evident initial manifestation of SLE and have highlighted our clinical approach for the same.

## CASE REPORT

**Figure 1:**
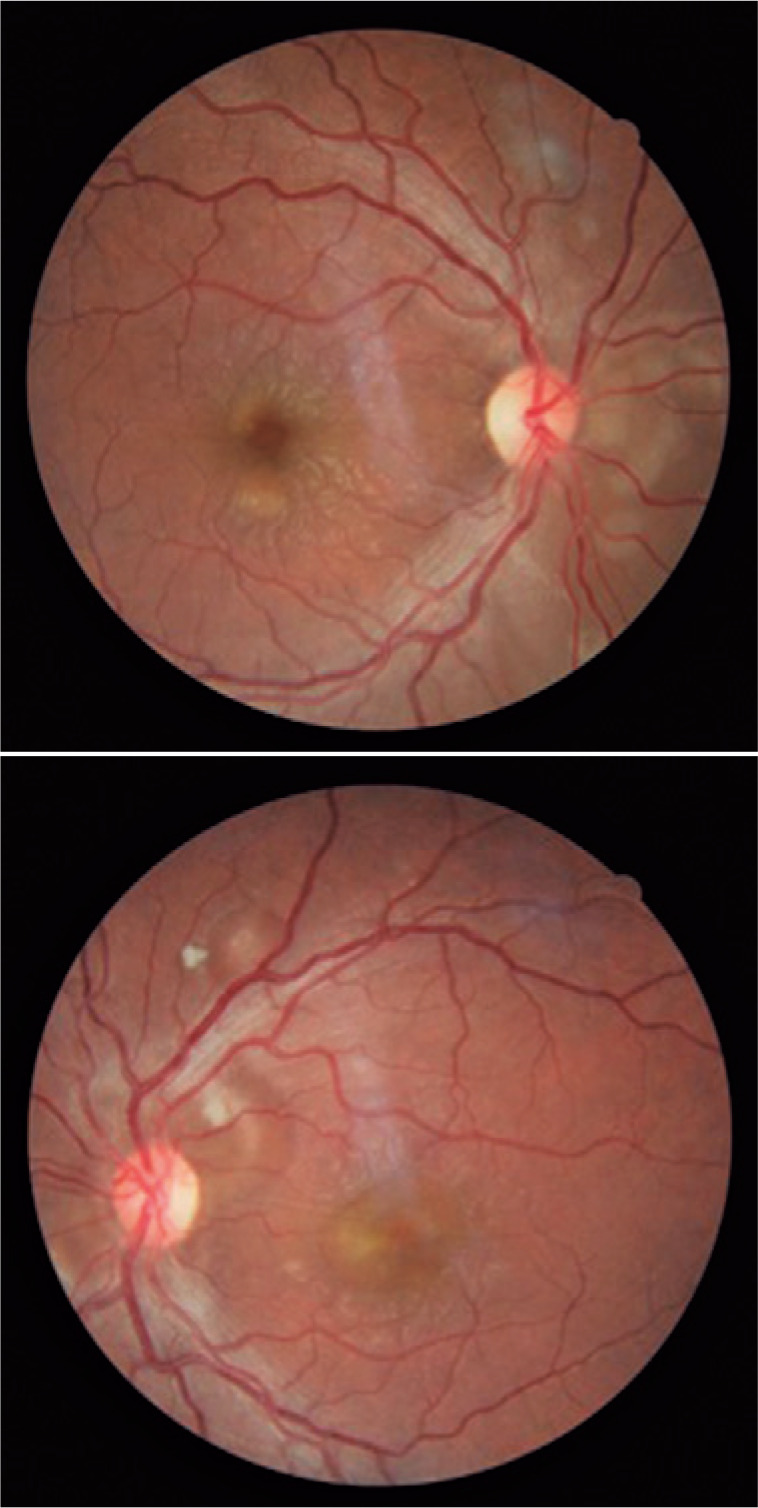
Fundus photograph showing bilateral retinal detachment around the disc and macular region in both the eyes in a patient with systemic lupus erythematosus

**Figure 2:**
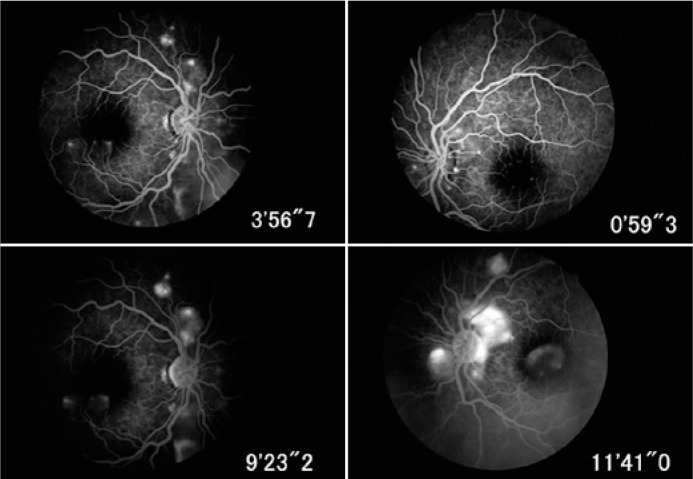
Fluorescein angiogram suggesting subretinal fluid pooling with multiple points of leakage at the retinal pigment epithelium in the corresponding serous retinal detachments in a patient with systemic lupus erythematosus

**Figure 3:**
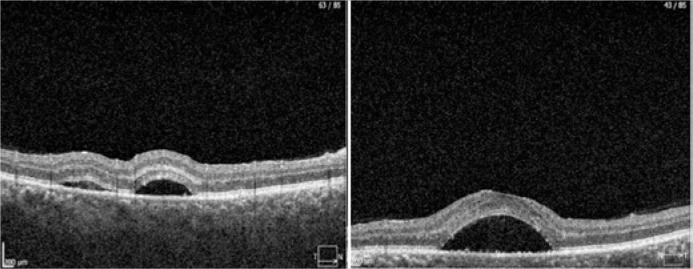
Optical coherence tomography (OCT) line B-scan of the macular region (fovea) showing subretinal fluid with some small hyper-reflective areas under the neural retina and above the retinal pigment epithelium in a patient with systemic lupus erythematosus. Right eye (right panel) and left eye (left panel)

A 23-year-old woman was referred to the ophthalmology center with the complain of bilateral progressive visual acuity (VA) loss since past 4 days. On ophthalmologic examination, her best corrected VA was 20/400 for both the eyes. Pupillary direct reflex was present, albeit weak, and there was no relative afferent pupillary defect, while the extraocular motility was normal. The anterior segment revealed no hyperemia, but remarkable chemosis, without anterior chamber cells or flare. The fundus examination revealed bilateral retinal detachment around the disk and macula with neither peripheral tears nor vitreous reaction (Figure 1). Fluorescein angiography (FA) demonstrated subretinal fluid pooling with multiple points of leakage at the retinal pigment epithelium (RPE) in the corresponding SRD (Figure 2). A B-scan ultrasound did not reveal posterior scleritis, such as retinochoroidal scleral thickening, T-sign, or sub-Tenon’s fluid. The optical coherence tomography (OCT) revealed subretinal fluid with small hyper-reflective areas under the neural retina and above the RPE (Figure 3). The subfoveal choroidal thickness was 276 OD and 277 OS. The ocular alterations were compatible with choroidopathy, and further investigation clarified the etiopatogenesis. The patient’s vital signs included a heart rate of 100, blood oxygen saturation of 91%, blood pressure of 110/50 mmHg, axillary temperature of 36.2^o^C, and normal respiratory rate. Her laboratory tests revealed hemoglobin level of 11.8 g/L, erythrocyte sedimentation rate (ESR) of 76, and serum albumin level of 2.2 g/dL. The results of daily proteinuria to investigate the hypoalbuminemia was 1,185.8 mg/24 h. The Coombs test results revealed autoimmune hemolytic anemia. The immunological profile indicated positive antinuclear antibodies (1/160). Due to the nephrological disorder, a renal biopsy was performed, which revealed a lupus nephritis type III, indicating SLE.

## DISCUSSION

The ophthalmic manifestation of SLE encompasses several ocular structures; of which retinal and choroidal involvement are vision-threatening. While the retinopathy caused by vasculitis or those secondary to hypertension is common and easy to diagnose due to the distinctive intraretinal hemorrhages, vascular sheathing, hard exudates, and disc edema, choroidopathy is a rare finding. The present case is notable because the SRD was the early manifestation of SLE that caused visual loss and required extensive investigation.

A wide variety of local and systemic pathologies cause secondary subretinal fluid accumulation, such as infectious diseases (primarily syphilis); inflammatory diseases (e.g., Vogt-Koyanagi-Harada, sympathetic ophthalmia, posterior scleritis, and Behçet’s); neoplastic conditions (including tumors, retinal lymphoma, Coats disease, melanoma, hemangioma, and leukemia); vascular conditions (e.g., venous occlusions, diabetic macular edema, and pre-eclampsia); and degenerative retinal diseases, including age-related macular disease, have also been associated with SRD^(3)^. Therefore, initially, epidemiological data and clinical history helped guide the diagnosis.

The present patient did not have hypertension, and hence we excluded malignant hypertension as the cause of SRD. Furthermore, there were no hemorrhages, disc edema, or other clinical features that could suggest any other possible causes. We hence considered central serous chorioretinopathy (CSC), even as an atypical ma nifestation in women^(4)^. Notably, acute bilateral detachment is an uncommon presentation of acute CSC, and, in the present case, it is difficult to distinguish it from SLE choroidopathy. Although we did not perform indocyanine green angiography, it is an important tool to detect choroidal changes and discriminate between these entities. Viana et al.^(5)^ studied the ICG and FA pattern in patients with chronic CSC, and demonstrated that, in the early phases, the ICG displays hyperfluorescent areas that do not appear in the FA due to increased choroidal permeability, which suggest that ICG is more sensitive than FA for detecting choroidal abnormalities in these cases. In contrast, Gharbiya et al.^(6)^ demonstrated that, in the early phase of ICG, patients with SLE choroidopathy presented with hypofluorescent areas that become hyperfluorescent in the intermediate to late phases. Thus, ICG can be considered to be essential to clarify our case.

Some cases of chronic CSC have been described simultaneously in patients with SLE. However, the retinal detachment could have been caused by the steroid therapy as well^(7)^. Another important tool to evaluate choroidopathies is the enhanced depth-imaging optical tomography coherence (EDI-OCT). This technology enables improved visualization of the choroid, and it has been shown that patients with CSC have an extremely thick choroid^(8)^. In our case, the history of acute visual loss and the presentation of normal choroidal thickness in the OCT led us to investigate other causes beyond chronic SCS.

Nevertheless, at the first ophthalmologic appointment, we inquired the patient, for a second time, regarding whether they were experiencing any other signs or symptoms. She showed us violet and scaly lesions on the palms of her hands and on the soles of her feet that suggested syphilis, which was however ruled out through infectious disease screening. At that point of time, the laboratory results suggested proteinuria, hemolytic anemia, and elevated ESR, which convinced us to question the patient again about other symptoms. The patient finally revealed painless oral ulcers and mild alopecia with cutaneous changes in the distal regions of progressive extremities that worsened under stress and cold conditions. Finally, these presentations led us to hypothesize an autoimmune cause, which indicated SLE. The examinations mentioned above in the case report thus concluded the diagnosis.

Although choroidal effusions are known to occur occasionally in SLE, only little is known about the mechanisms that lead to fluid accumulation in the suprachoroidal space^(9)^. The inflammatory lesions in a different part of the body, which is a characteristic of SLE, are accompanied by immunoglobulins and complement components that are deposited in a granular pattern, suggesting immune complex aggregates. Stefater et al.^(10)^ suggested that choroidal effusions in acute SLE are probably related to localized choroidal inflammation. It is therefore possible that the choroid is a primary target for the deposition of immune complexes and that it does not simply respond to low oncotic pressure. The etiology of nephropathy is believed to be related to the deposition of immune complexes with localized inflammation and glomerular dysfunction.

Lupus choroidopathy is considered as a systemic disease-activity marker, and, in the present case, it was the primary manifestation. The presence of choroidopathy indicates aggressive and prolonged immunosuppression, which requires monitoring by an ophthalmologist and rheumatologist^(2)^.

The fact that SRD was presented as an initial manifestation of SLE can be considered as a diagnostic challenge for patients who do not have other clear systemic symptoms of SLE.
